# Tripolyphosphate-Crosslinked Chitosan/Gelatin Biocomposite Ink for 3D Printing of Uniaxial Scaffolds

**DOI:** 10.3389/fbioe.2020.00400

**Published:** 2020-04-30

**Authors:** Tiziana Fischetti, Nehar Celikkin, Nicola Contessi Negrini, Silvia Farè, Wojciech Swieszkowski

**Affiliations:** ^1^Faculty of Materials Science and Engineering, Warsaw University of Technology, Warsaw, Poland; ^2^Department of Chemistry, Materials and Chemical Engineering “G. Natta”, Politecnico di Milano, Milan, Italy; ^3^INSTM, National Consortium of Materials Science and Technology, Local Unit Politecnico di Milano, Milan, Italy

**Keywords:** chitosan, gelatin, 3D printing, ionic crosslinking, uniaxial tissue engineering, scaffolds

## Abstract

Chitosan is a natural polymer widely investigated and used due to its antibacterial activity, mucoadhesive, analgesic, and hemostatic properties. Its biocompatibility makes chitosan a favorable candidate for different applications in tissue engineering (TE), such as skin, bone, and cartilage tissue regeneration. Despite promising results obtained with chitosan 3D scaffolds, significant challenges persist in fabricating hydrogel structures with ordered architectures and biological properties to mimic native tissues. In this work, chitosan has been investigated aiming at designing and fabricating uniaxial scaffolds which can be proposed for the regeneration of anisotropic tissues (i.e., skin, skeletal muscle, myocardium) by 3D printing technology. Chitosan was blended with gelatin to form a polyelectrolyte complex in two different ratios, to improve printability and shape retention. After the optimization of the printing process parameters, different crosslinking conditions were investigated, and the 3D printed samples were characterized. Tripolyphosphate (TPP) was used as crosslinker for chitosan-based scaffolds. For the optimization of the printing temperature, the sol-gel temperature of the chitosan-gelatin blend was determined by rheological measurements and extrusion temperature was set to 20°C (i.e., below sol-gel temperature). The shape fidelity and surface morphology of the 3D printed scaffolds after crosslinking was dependent on crosslinking conditions. Interestingly, mechanical properties of the scaffolds were also significantly affected by the crosslinking conditions, nonetheless the stability of the scaffolds was strongly determined by the content of gelatin in the blend. Lastly, *in vitro* cytocompatibility test was performed to evaluate the interactions between L929 cells and the 3D printed samples. 2% w/v chitosan and 4% w/v gelatin hydrogel scaffolds crosslinked with 10% TPP, 30 min at 4°C following 30 min at 37°C have shown cytocompatible and stable characteristics, compared to all other tested conditions, showing suitable properties for the regeneration of anisotropic tissues.

## Introduction

Chitosan is a natural polysaccharide derived from the partial deacetylation of chitin, a polymer present in the exoskeleton of crustaceans, insects and fungi (Elieh-Ali-Komi and Hamblin, 2016). The chitosan chemical structure is constituted by D-glucosamine and N-acetyl-D-glucosamine linked by β-(1-4) glycosidic bonds, in which the glucosamine is the main repeating unit. Amino groups present in D-glucosamine can be protonated in acidic aqueous solutions (pH < 6), bringing to the formation of a polycationic polymer (Croisier and Jérôme, 2013). For its polycationic behavior, chitosan can form ionic complexes with different anionic species, both deriving from natural or synthetic sources, as lipids, proteins, DNA, polystyrenesulphate, oleate and dextran sulfate (Rinaudo, 2006; Chen et al., 2007; Kim et al., 2008; Schwarz et al., 2012; Ng et al., 2016). Chitosan exhibits other intrinsic properties thanks to its polycationic nature, such as antibacterial (Ong et al., 2008) and antifungal activity (Aranaz et al., 2009), and mucoadhesive (Sogias et al., 2008), analgesic (Aranaz et al., 2009) and hemostatic properties (Ong et al., 2008). Chitosan is abundantly used in biomaterials science, food industry, biomedical, and pharmaceutical applications (Ravi Kumar, 2000; Rinaudo, 2006) and it offers the advantage of being easily processable into gels (Huang et al., 2011), membranes (Lin et al., 2013), nanofibers (Jayakumar et al., 2010), beads (Buranachai et al., 2010), nanoparticles (Moreira et al., 2016; Thandapani et al., 2017), scaffolds (Jana et al., 2013; Rodríguez-Vázquez et al., 2015), and porous foams (Ji et al., 2011).

Anisotropic tissues enable highly elaborate functions of living organisms (Haque et al., 2010). When these tissues are impaired by pathological conditions or trauma, their regeneration is challenging and it has been poorly investigated. Tissue engineering (TE) scaffolds for anisotropic tissues have to satisfy the requirement of creating regional and directional anisotropy in three-dimensional space. To obtain this, the design and obtainment of patterns able to induce cell orientation along one preferential direction is needed, together with the enhancement of the mechanical properties in this direction (De France et al., 2017). The most used techniques to generate anisotropy *in vitro* are the use of mechanical force (Haque et al., 2010; Nardinocchi and Teresi, 2016), directional freeze-casting (Chen et al., 2012; Bai et al., 2013) and micro-patterning (Li et al., 2014). In recent years, 3D printing technology has been also considered as valid alternative to fabricate anisotropic patterns for TE. This technique exploits the use of a 3D CAD which is later converted into a code, and manufacturing of a 3D construct with desired architecture is then obtained (Murphy and Atala, 2014). The main advantages of 3D printing are the automation and reproducibility of the process, with the precise deposition control of the scaffold structure based on the 3D model. Additionally, the possibility to obtain 3D printed physical constructs based on clinical images (e.g., CT, MRI) allows obtaining patient specific scaffolds, which makes 3D printing particularly useful for TE.

Despite 3D printing technology has been extensively investigated for TE applications, materials choice and optimization of printing parameters for defined micro-architectures are still compelling (Chia and Wu, 2015). Chitosan-based scaffolds for skin (Ng et al., 2016), bone (Demirtaş et al., 2017), and cartilage regeneration (Ye et al., 2014) fulfill the needed requirements in terms of cell viability (Elviri et al., 2017; Pollot et al., 2017); however, chitosan possesses poor printability and weak mechanical properties (Ng et al., 2016). For this reason, 3D printing of pristine chitosan is extremely challenging, and further modifications are required to increase the printability of chitosan-based scaffolds (Ng et al., 2016; Demirtaş et al., 2017). For example, gelatin can be used to improve the printability and shape fidelity of the printed construct (Golden and Tien, 2007; Neal et al., 2014; Hinton et al., 2015; Ng et al., 2016).

In this study, gelatin is blended with chitosan to improve the printability and shape fidelity of the printed constructs. The rheological characteristics of chitosan – gelatin blend with different chitosan/gelatin ratios are investigated. The printing process in terms of printing parameters (i.e., temperature, extrusion pressure, deposition speed) is optimized. Different crosslinking conditions are tested for the 3D printed constructs (0–180° fiber configuration), characterized through the coherence of printed fiber diameter to CAD design, mechanical and stability properties. The suitability of the scaffolds for anisotropic TE is evaluated by a preliminary *in vitro* cytocompatibility test. Through the abovementioned characterizations and evaluations, here, we propose the use of chitosan – gelatin blends as biomaterial ink and tripolyphosphate (TPP) as crosslinker to print uniaxial 3D scaffolds, for the first time.

## Materials and Methods

### Materials

Chitosan (low molecular, 50–190 kDa, 75–85% deacetylation), gelatin (bovine skin, type B), sodium tripolyphosphate (TPP), and phosphate buffered saline (PBS) were purchased from Sigma Aldrich.

### Preparation of CHIGEL Hydrogel Blends

Chitosan-gelatin (CHIGEL) hydrogel blends were prepared in two different chitosan:gelatin ratios (1:2 and 1:3 w:w), identified in the following as 1CHI2GEL and 1CHI3GEL. For their preparation, 3% (w/v) chitosan was dissolved in 2% (v/v) acetic acid under magnetic stirring for 3 h at room temperature. Gelatin, 12% (w/v) and 18% (w/v), was dissolved in PBS and stirred at 40°C. Before forming the chitosan-gelatin blend, the pH of 3% chitosan solution was increased to 4.7 by addition of 0.5 M NaOH; gelatin, at this pH value, starts to form the polyelectrolyte complex between the negative charges of gelatin and the positive charges of chitosan. Consequently, gelatin was added to chitosan solution, and the pH of the chitosan-gelatin blend was adjusted to 6.5 by addition of NaOH. Indeed, at this pH (i.e., 6.5), chitosan amino groups are deprotonated and form insoluble weak chitosan polymer.

### Rheological Characterization

The rheological properties of the hydrogel blends were evaluated using ARES rheometer (TA Instruments, New Castle, DE, United States) with cone-plate geometry (diameter 50 mm, cone angle 0.1 rad), setting the gap at 50 μm. An oscillatory strain sweep test was firstly performed, with strain range 0–10% and frequency 1 Hz. This test was performed to study the values of the strain amplitude within the linear viscoelastic region (LVR); the shear strain value was chosen equal to 5%. To evaluate the sol-gel transition temperature, storage modulus (G’) and loss modulus (G”) of 1CHI2GEL and 1CHI3GEL were measured in the temperature range 4–40°C, with an increasing temperature ramp of 2°C/min. The complex viscosity (η^∗^) of 1CHI2GEL and 1CHI3GEL was investigated for shear rates ranging from 0.1 to 100 s^–1^ at 20°C (i.e., optimized printing temperature).

### Optimization of 3D Printing Process and Crosslinking of CHIGEL Scaffolds

A 3D Bioplotter (EnvisionTEC GmbH, Germany) was used to fabricate 3D printed scaffolds using an extrusion-based 3D printing technique. 3D printed scaffolds were designed to have dimensions corresponding to 12 mm × 10 mm × 2 mm. The scaffolds were printed with a 23G (inner diameter, ID = 330 μm) needle. Printing parameters were evaluated in the range 1–4 bar for the extrusion pressure and 2–30 mm/s for the deposition speed. After the optimization process, the parameters were set to 2.5 bar for the extrusion pressure and to 10 mm/s for the deposition speed. The obtained 3D printed scaffold was constituted by five layers, each formed by parallel superimposed fibers. The fiber orientation in adjacent layers was set to 0–180°, to ease the mimic of the anisotropic tissue architecture in longitudinal direction. Distance between the fibers was set to 1 mm, as for lower values fibers joined between each other, due to partial fiber collapse. The value of layer thickness (LT) was set at 250 μm, as from literature the ideal value of LT is the 80% of the needle size dimension, to favor the surface contact between consecutive deposited layers (Mozetic et al., 2017). The temperature during the printing process, previously assessed by rheological characterization, was set at 20°C, to have the CHIGEL in a gel state. The temperature of the base plate was set at 4°C, to guarantee the maintenance of the shape during the printing process, due to the gelation of gelatin at low temperatures (i.e., T < T_sol–gel_).

After the printing process, the printed scaffolds were immersed in 10% w/v TPP crosslinker. The overall crosslinking time of the 3D printed structure was set to 60 min, due to chitosan slow gelation rate with TPP (Gan et al., 2005). The following three different crosslinking conditions were tested: (i) 3D printed structure kept at 4°C for 60 min with TPP, (ii) 3D printed structure kept at 4°C for 30 min and moved to 37°C for the remaining 30 min, or (iii) 3D printed structure kept at 4°C for 10 min and moved to 37°C for the remaining 50 min. The two selected temperatures, 4 and 37°C, in which CHIGEL blend exhibits a gel and solution state, respectively, were chosen to evaluate the thermo-sensitive behavior of the blends and to detect any differences within the considered conditions, for different exposition time. In all these cases, after 60 min of TPP crosslinking, TPP solution was removed and replaced with PBS, to remove possible unreacted residuals. The identification acronyms for the considered chitosan/gelatin structures are summarized for each ratio and crosslinking condition in Table 1.

**TABLE 1:** Initial and final concentrations of chitosan (*) and gelatin (**) used to form 1:2 and 1:3 Chitosan: Gelatin blend ratios (identified as 1CHI2GEL and 1CHI3GEL, respectively).

	Initial concentrations	Final concentrations	Identification name	Crosslinking condition		
				
				60_0	30_30	10_50
*Chitosan-**Gelatin	*3%w/v-**12%w/v	*2%w/v-**4%w/v	1_2 Chito_Gel	1CHI2GEL 60_0	1CHI2GEL 30_30	1CHI2GEL 10_50
	*3%w/v-**18%w/v	*2%w/v-**6%w/v	1_3 Chito_Gel	1CHI3GEL 60_0	1CHI3GEL 30_30	1CHI3GEL 10_50

*In the last column on the right, the coding system declined for each ratio used (1CHI2GEL, 1CHI3GEL) and crosslinking condition (60_0,30_30,10_50) is reported.*

### Scanning Electron Microscopy (SEM) Observation

The morphology of the 3D printed structures for each crosslinking condition and ratio was observed by SEM (Table 1). Briefly, the samples were dehydrated in a graded ethanol series concentration (50, 70, 80, 90, 100%, 10 min for each) and then with hexamethyldisalazane (HDMS). To prepare samples for SEM observation, scaffolds were sputtered with 7 nm gold and the morphology was observed with proX desktop (Phenom) SEM at 10 kV, with different magnifications (285X, 1850X, 4600X).

### Fourier Transform Infrared Spectroscopy (FTIR)

Fourier transform infrared spectroscopy analysis was performed on pristine chitosan and gelatin powders, and CHIGEL blends (Table 1) constituting the 3D printed structures. FTIR analysis was performed to detect the possible interactions between chitosan and gelatin in the considered blends, and to attain the differences among the crosslinking conditions and ratio (Table 1). Analyses were performed with Q5000 FTIR (TA Instruments, New Castle, DE, United States); the spectra were recorded in absorbance mode in the 4000–400 cm^–1^ range, with a 4 cm^–1^ resolution and 128 scans.

### Mechanical Characterization

Compression mechanical tests were performed on the 3D printed scaffolds for each ratio and crosslinking condition (Table 1) using a dynamic mechanical analyzer (DMA Q800, TA Instrument, New Castle, DE, United States) in unconfined compression mode to test the suitability of the obtained structures in mimicking the mechanical properties of anisotropic tissues. Samples (*n* = 3, 12 mm × 10 mm × 2 mm), were incubated at 37°C in 2 ml of 0.02% w/v sodium azide solution for 48 h, and then tested. Tests were performed in strain-controlled mode at 37°C, after a 5 min isotherm at 37°C, by applying a compressive strain rate of 2.5% min^–1^ down to −30% strain (0.001 N preload force). Then, the compression force was removed, and an unload phase was performed at 5% min^–1^. The following mechanical parameters were evaluated from the obtained stress-strain curves: elastic modulus (calculated as the slope of the curve in the 0–5% range, E_0__–__5__%_), stiffness at maximum load (calculated as the slope of the curve in the 25–30% range, E_25__–__30__%_), maximum stress (calculated as the stress corresponding to 30% strain, σ_max_), residual strain (calculated as the strain corresponding to null strain at the end of the unload phase ε_res_), and hysteresis area (calculated as the area between the loading and unloading curves, H) (Negrini et al., 2020).

### Shape Fidelity Characterization

The shape fidelity of the 3D printed samples was investigated for each ratio (1CHI2GEL, 1CHI3GEL) and crosslinking condition (60_0, 30_30, 10_50). Measurements were acquired immediately after the crosslinking time-period, considering the dimensions of the printed samples (i.e., length, width, height), fiber diameter, and distance between fibers. For each ratio and crosslinking condition considered in this study, the measurements of samples (*n* = 4) were obtained. As regarding the fiber dimensions, the diameter of each fiber for each condition was measured at seven different points and averaged. The same was performed for the measurements of the distance between the fibers, defined as the distance between the axes of two adjacent fibers (Supplementary Figure S1). The dimensions were acquired with an optical microscope (Leica TCS SP8). Quantitative measurements for the different conditions were compared to the theoretical ones, using the equation (Eq. 1):

(1) $$\mathrm{Accurancy}\left[\%\right]=\frac{100}{n}*\sum_{1}^{n}\left(1-\frac{\left|\mathrm{dr}-\mathrm{ds}\right|}{\mathrm{ds}}\right)$$

where *d*_*r*_ is the measured sample dimension, *d*_*s*_ the theoretical dimension (i.e., *d*_s_ = 12 mm, 10 mm, 1,25 mm, 330 μm, and 1 mm for length, width, height, needle size, and distance between fibers, respectively), *n* is the number of the considered samples (*n* = 4 × 7 different points).

### Stability Test

Stability test was performed on the samples of each ratio and each evaluated crosslinking condition to evaluate the residual weight (RW%) of samples after immersion in water at 37°C. Samples (*n* = 5 for each condition) were weighted in wet condition, i.e., immediately after the crosslinking (W_0_). Consequently, samples immersed in 2 ml of 0.02% w/v sodium azide in distilled water at 37°C. At established time points (*t* = 1, 3, 24, 48 h, 3, 7, 14, 21 days) samples were removed from the solution, gently swabbed with tissue paper and weighted (W_t_). Residual weight (*RW*%) was calculated with the following equation (Eq. 2):

(2) $$\mathrm{RW}\%=\frac{W_{t}}{W_{0}}\times 100$$

### *In vitro* Cytocompatibility of the CHIGEL Scaffolds

Preliminary *in vitro* cytocompatibility test was performed using L929 fibroblasts cell line to test cytocompatibility of the materials constituting the 3D structures and the different crosslinking conditions performed. After the physico-chemical and mechanical characterization, only 1CHI2GEL ratio and 30_30, 10_50 crosslinking conditions were considered. To prevent any contamination, gelatin and chitosan powders were sterilized by UV light (λ = 100–280 nm) for 30 min. The 3D structures (12 mm × 10 mm × 2 mm) were printed in sterile condition, then washed in sterile PBS overnight to remove potential residues and put in contact with L929 cells (from mouse C3H/An, ECACC, United Kingdom). For each tested condition, *n* = 4 replicates were considered. Positive control was constituted by L929 cells cultured in complete DMEM and 0.1% Triton X100 (i.e., dead cells), and negative control was constituted by L929 cells cultured in complete DMEM (i.e., live cells), representing the worst and the optimal cells viability condition, respectively. 3D printed CHIGEL scaffolds were seeded with 3 × 10^4^ cells/well, and cultured in Dulbecco’s Modified Eagle Medium (DMEM, Gibco, Grand Island, MA, United States) supplemented with 10% FBS (EuroClone S.p.A., Pero, Italy), and 100 μg/mL Penicillin-Streptomycin (10,000 U/mL, Gibco) at 37°C, 5% CO_2_, up to 72 h. Subsequently, 24 and 72 h after seeding, CellTiter Cell Proliferation Assay (MTS, Promega, Fitchburg, WI, United States) was performed to evaluate the metabolic activity (FLUOstar Omega UV/Vis spectrometer, BMG LabTech, Ortenberg, Germany) of fibroblasts on the 3D printed CHIGEL hydrogels. Metabolic activity was evaluated by UV spectrophotometer (FLUOstar Omega), considering the absorbance at λ = 490 nm. Percentage cell viability was calculated by using the following equation (Eq. 3):

(3) $$\mathrm{Cell}\mathrm{Viability}\left(\%\right)=\frac{A_{S}-A_{P}}{A_{N}-A_{P}}*100$$

where *A*_*S*_,*A*_*P*_, and *A*_*N*_ are the sample absorbance, the positive control absorbance, and the negative control absorbance, respectively. Cytocompatibility results were reported in terms of cell viability (%) and compared to the negative control (i.e., cells in complete DMEM seeded on tissue culture plastic).

### Statistical Analysis

Results are expressed as mean ± standard deviation. One-way ANOVA with Tukey’s multiple comparison tests were performed using GraphPad Prism software to investigate statistical difference between data populations. A *p*-value (*p*) < 0.05 was considered as statistically significant.

## Results and Discussion

### Rheological Characterization and Printing Temperature Optimization

**FIGURE 1:**
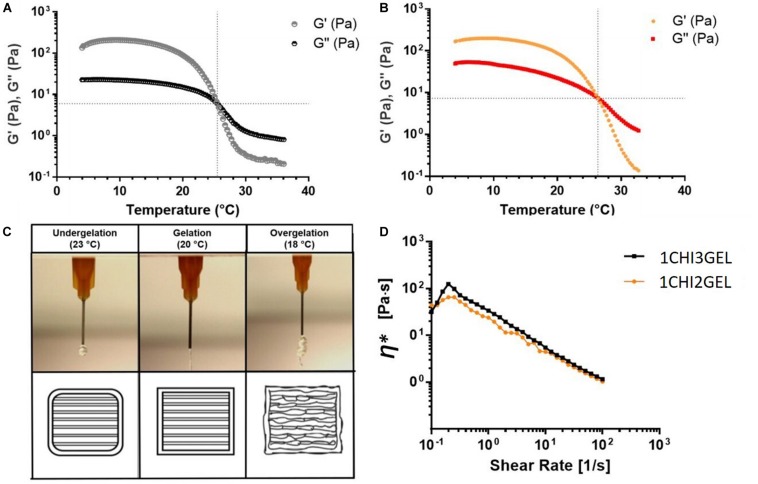
Rheological characterization of chitosan – gelatin blends. Storage (G′) and loss (G″) moduli at varying temperatures for **(A)** 1CHI2GEL, and **(B)** 1CHI3GEL. **(C)** Extrusion of the gel at varying temperatures (18, 20, 23°C). **(D)** Complex viscosity η* behavior in function of the shear rate for 1CHI2GEL and 1CHI3GEL.

The rheological characterization for 1CHI2GEL, 1CHI3GEL was performed in the range 4–40°C, to determine the sol-gel transition temperature (T_sol–gel_) of the two blends. T_sol–gel_ was detected at 25.5 ± 1.0°C for 1CHI2GEL (4% w/v gelatin, Figure 1A), and at 26.4 ± 0.9°C for 1CHI3GEL, (6% w/v gelatin, Figure 1B). The results showed that the chitosan–gelatin blends were in a gel state below the gel point temperatures, and in a liquid-like state above them.

To optimize printing temperature, extrusion at different temperatures (T = 18, 20, 23°C), all below the T_sol–gel_ transition (T = 25.5 and 26.4°C), were evaluated (Figure 1C) to ensure the shape fidelity when gelatin is in the gel state. For both 1CHI2GEL and 1CHI3GEL blends at 18°C, the extrusion was not smooth; in fact, irregular fragments were obtained. At 20°C, the extrusion was continuous, and it was possible to obtain smooth fibers. On the contrary, at 23°C, temperature closer to the T_sol–gel_, the gel was weak and not able to maintain a good shape fidelity. Thus, 20°C was selected as the temperature to extrude the gels during the printing process, for both the CHIGEL ratios considered in the study. At this temperature, the complex viscosity η^∗^ of 1CHI2GEL and 1CHI3GEL, in function of the shear rate (Figure 1D), was investigated. For both the blends (1CHI2GEL and 1CHI3GEL), a slightly increase in η^∗^ was observed up to shear rate values equal to 0.2 s^–1^; in fact, the gels had to overcome the yield stress point at this value. Then, a continuous decrease in viscosity for both the ratios was detected, until 1 Pa⋅s at 100 s^–1^. The decrease in viscosity with increasing shear rate (i.e., shear thinning response) is the typical behavior of non-Newtonian fluids. Hydrogels showing shear thinning behavior are particularly suitable in extrusion-based 3D printing systems (Costantini et al., 2016; Ng et al., 2016); indeed, they are characterized by a decrease in viscosity under applied shear, as it occurs during the extrusion of the gel through the needle in the 3D printing process.

### Optimization of the Printing Process

**FIGURE 2:**
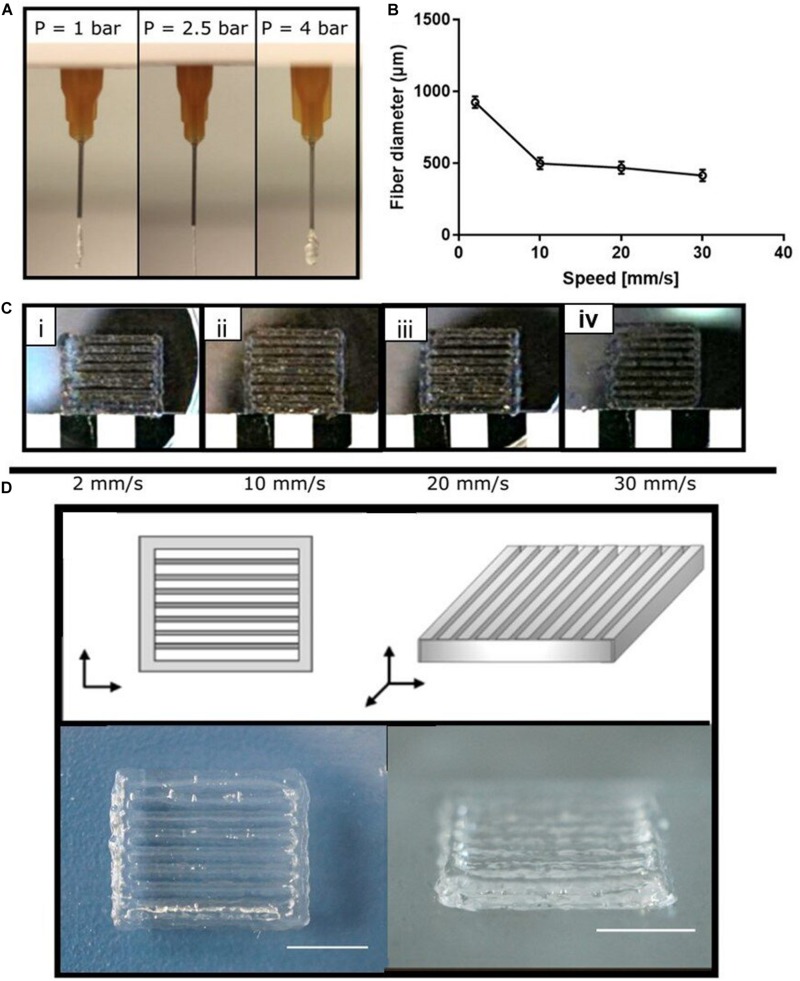
Optimization of the chitosan-gelatin blend printing parameters. **(A)** Extrusion of the blend at different pressures. **(B)** Printed fiber diameter at different speed values. **(C)** Effect of the combination between the selected pressure (*P* = 2.5 bar) and different speed values; **(D)** on the top panel schematic representation of the 3D designed scaffold and on the lower panel frontal and lateral view of the obtained 3D printed scaffold (i.e., 1CHI2GEL, 2% w/v chitosan, 4% w/v gelatin).

After the rheological characterization and the selection of the printing temperature (T = 20°C), the optimization of the printing parameters (i.e., extrusion pressure and deposition speed) was attained. The optimal combination of these parameters was selected as the one allowing for the continuous extrusion of structurally stable 3D structures. First, the extrusion pressure was optimized (range 1–4 bar). In particular, 1, 2.5, and 4 bar pressure values were considered (Figure 2A). *P* = 1 bar was not sufficient to extrude the hydrogel through the needle; *P* = 2.5 bar allowed for the extrusion of a continuous and steady fiber; *P* = 4 bar resulted in dispersion of cluster of material. After the selection of the optimal extrusion pressure (*P* = 2.5 bar), this was combined with different values of deposition speed (range 2–30 mm/s). In particular, 2, 10, 20, 30 mm/s deposition speed values were considered. In Figure 2B, single fiber diameters were reported as the extrusion speed varies. In particular, the fiber diameter decreased for increasing speed values; in fact, by increasing the speed values, the 3D printer does not have enough time to deposit the hydrogel fiber, resulting in progressive fiber thinning or lack of deposition on the surface of the platform (Ng et al., 2016). Combining the selected pressure (*P* = 2.5 bar) with the different values of deposition speed, it was noticed that for *P* = 2.5 bar and speed = 2 mm/s, merged fibers were obtained (Figure 2C_i). By increasing speed, a shear thinning of the fibers in some points (speed = 20 mm/s, Figure 2C_iii) and lack of the deposited hydrogel in other points (speed = 30 mm/s, Figure 2C_iv) were observed. The optimal values that allowed detecting all the deposited fibers and obtaining continuous deposition of the hydrogels were found for *P* = 2.5 bar and speed = 10 mm/s (Figure 2C_ii). The optimized printing parameters are summarized in Table 2, and the 3D printed structure obtained with the optimized parameters is shown in Figure 2D. The use of the optimized printing parameters allowed obtaining comparable results for the different chitosan/gelatin ratio considered in this study (i.e., 1CHI2GEL and 1CHI3GEL). The higher gelatin content in the 1CHI3GEL, resulting in higher viscosity (i.e., higher number of interactions between the positively charged amine groups from chitosan and the negatively charged carboxylate groups from gelatin) did not significantly affect the gel extrusion. Indeed, the viscosity values reported for 1CHI2GEL and 1CHI3GEL are very close between each other (Figure 1D) and overlap from 10 to 100 s^–1^, demonstrating the possible use of the same printing parameters (*P* = 2.5 bar, speed = 10mm/s).

**TABLE 2:** Optimized printing parameters for the chitosan-gelatin blend.

Parameters	Values
Cartridge temperature [°C]	20
Plate temperature [°C]	4
Pressure [bar]	2.5
Speed [mm/s]	10
Layer thickness [μm]	250
Distance between fibers [mm]	1
Fiber orientation	0/180°

### Crosslinking Effects on 3D Printed Structures

#### Post Crosslinking Processing

**FIGURE 3:**
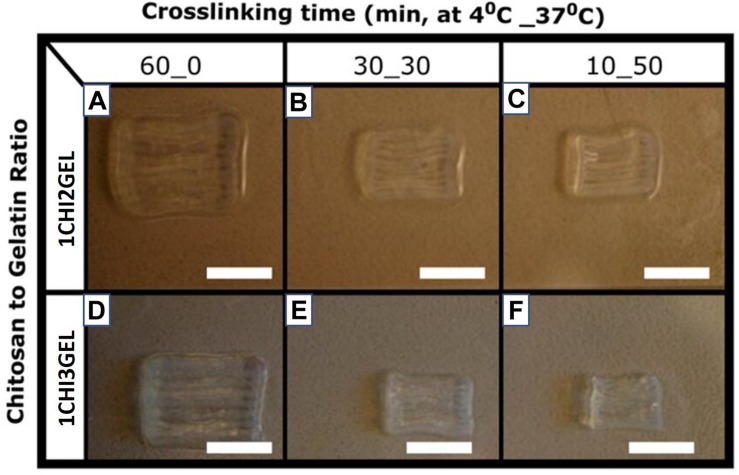
Printed structure obtained for each ratio (1CHI2GEL on the top, and 1CHI3GEL on the bottom) and for each crosslinking condition performed, respectively, 60_0, 30_30, and 10_50. Scale bar: 5 mm. From a qualitative point of view, **(A,D)** 60_0 crosslinking condition shows the best results in terms of shape integrity, while **(B,E)** 30_30 and **(C,F)** 10_50 lose the structural integrity.

The 3D printing of chitosan scaffolds was possible through formation of a polyelectrolyte complex between chitosan and gelatin, as the latter works as thickener and support material (Piard et al., 2019). However, to ensure the stability of the 3D printed structure after 3D printing process and to be able to handle the scaffolds without losing their structural integrity, 10% w/v TPP crosslinker was used as crosslinker. TPP was poured on the 3D printed structure immediately after the printing process. TPP has been previously used to crosslink chitosan beads (Buranachai et al., 2010), nanoparticles (Gan et al., 2005; Thandapani et al., 2017), and films (Liao and Ho, 2011); however, the use of TPP to crosslink 3D chitosan-based printed scaffolds has been poorly investigated (Serra et al., 2015). TPP crosslinker could represent an optimal choice and substitution to other chitosan crosslinkers, such as glutaraldehyde and genipin. Indeed, glutaraldehyde, the most common agent used to crosslink chitosan, may have a cytotoxic effect on cells if its residues are not completely eliminated (Liao and Ho, 2011; Serra et al., 2015). Genipin, a natural compound that shows a significantly lower level of cytotoxicity compared to glutaraldehyde, is a highly priced reagent (Gattazzo et al., 2018). In this work, TPP crosslinking has been performed at different temperatures, 4 and 37°C (Table 1). Due to gelatin stability at 4°C, 60_0 crosslinking condition shows the best results in terms of shape integrity and retention just after the crosslinking (Figures 3A,D). On the contrary, as the gelatin has predominant liquid-like behavior at 37°C, for 10_50 and 30_30 crosslinking conditions, the structures tend to collapse and lose the retention (Figures 3B,C,E,F).

#### FT-IR Analysis

**FIGURE 4:**
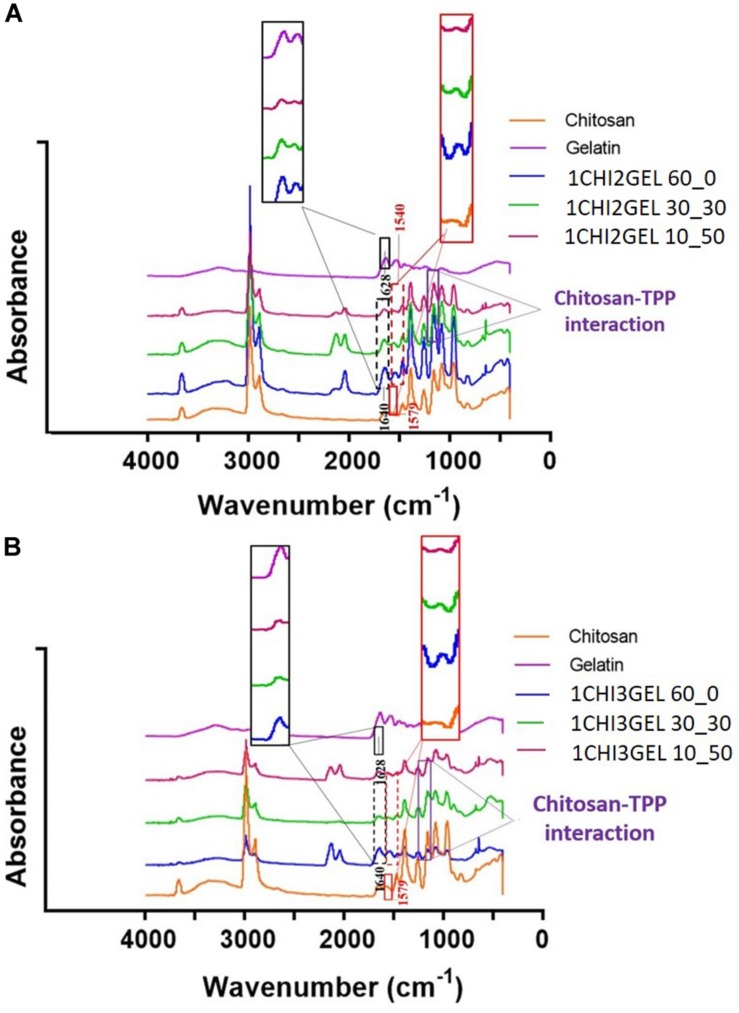
FTIR characterization of 3D printed chitosan- gelatin scaffolds. **(A)** FT-IR spectra of both pure chitosan and gelatin material, and different crosslinking conditions for 1CHI2GEL. The black and red boxes report a magnification of the shifts in the blends spectra, compared to the pristine material spectra. **(B)** FT-IR spectra of both pure chitosan and gelatin material, and different crosslinking conditions for 1CHI3GEL.

The interactions between chitosan and gelatin within the blend and the effect of the TPP crosslinking for each crosslinking condition (Table 1) were evaluated by FT-IR analysis. FT-IR spectrum of pristine chitosan and gelatin powders were considered as controls. Chitosan exhibited polysaccharide peaks at approximately 815 and 1151 cm^–1^. The peaks at 1251 cm^–1^, 1579 cm^–1^, and 1647 correspond, respectively, to C-N and N-H vibrations in amide III, to N-H and C-N vibrations in amide II and to C = O and N-H vibrations in amide I (the peak of the acetyl group) (Table 3). The IR spectrum of gelatin is characterized by peaks at 1235 cm^–1^, 1524 cm^–1^, 1628 cm^–1^, 3071 cm^–1^, corresponding respectively, to C-N, N-H vibrations in amide III, to N-H and C-N vibrations in amide II, to C = O and N-H vibrations in amide I (peak of carbonyl group), and to N-H vibrations in Amide A (Table 3). From the comparison between the spectra of the pristine materials and the ones obtained from the blend for each crosslinking condition, some shifts and disappearance of some peaks were detected (Figures 4A,B). In particular, in all the FTIR spectra acquired for CHIGEL, for each ratio and crosslinked condition, it was observed that the carbonyl groups shifted from 1628 cm^–1^ to 1640 cm^–1^, and the amino groups shifted from 1579 cm^–1^ to 1540 cm^–1^. It was also noticed that the gelatin peak at 2934 cm^–1^ attributed to amide B, and the one at 1397 cm^–1^, related to amide III, disappeared in the blend, due to the chitosan-gelatin electrostatic interactions (Table 3).

**TABLE 3:** FT-IR main chemical groups of chitosan, gelatin, and chitosan-gelatin blend.

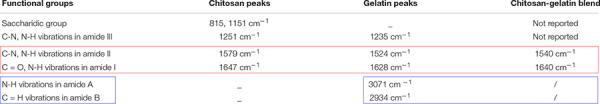

*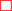 Shift in the chitosan-gelatin blend, 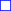 Peak disappearance (/) in the chitosan-gelatin blend.*

Similar results were reported in previous FT-IR spectra performed on chitosan-gelatin blend (Hajiabbas et al., 2015; Ng et al., 2016). Besides the interaction between chitosan and gelatin within the blend, the peak at 1151 cm^–1^ (evidenced with the purple rectangle in Figure 4) could also be hypothesized to represent the interaction between TPP crosslinker, as reported in previous works (Gan et al., 2005; Bhumkar and Pokharkar, 2006). However, this interaction could not be detected in the spectra, due to the overlapping with the saccharide peak of chitosan. Within the spectra of the blends for each ratio (Figure 4A for 1CHI2GEL, Figure 4B for 1CHI3GEL) and crosslinking condition, no differences were detected, as the interacting groups were the same.

#### SEM Morphology

**FIGURE 5:**
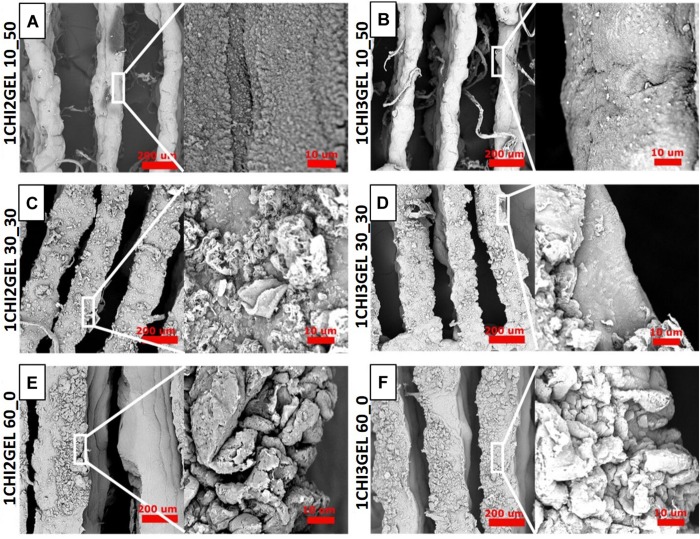
Morphological characterization of 3D printed chitosan-gelatin scaffolds. **(A–F)** SEM morphology of both the ratio and the crosslinking conditions (60_0, 30_30, 10_50) at different magnifications (Scale bar: 200 μm, 10 μm).

The surface morphology of 1CHI2GEL and 1CHI3GEL for each crosslinking condition (60_0, 30_30, 10_50) was investigated by SEM (Figures 5A–F). Qualitative differences can be detected between the 60_0 (Figures 5E,F) and both the 30_30 (Figures 5C,D) and 10_50 (Figures 5A,B) crosslinking conditions, for both the considered blends. The surface morphology of the 60_0 scaffold was rougher compared to the others, where this difference became more evident between the 60_0 and the 10_50 structures. The roughness characterizing the 60_0 condition of crosslinking that was performed at 4°C could be attributed to the presence of gelatin, which is in the gel state at low temperatures. In 30_30 and 10_50 conditions, the roughness effect is reduced, as the samples were maintained at 37°C, temperature at which gelatin is in liquid state. It is likely that, at 37°C, gelatin dissolves and a coating-like structure is generated on the 3D printed sample surface, giving it a more homogenous aspect. This morphological difference is more evident in 10_50 condition compared to the 30_30, as the first is maintained at 37°C for longer time.

#### Compressive Mechanical Characterization

**FIGURE 6:**
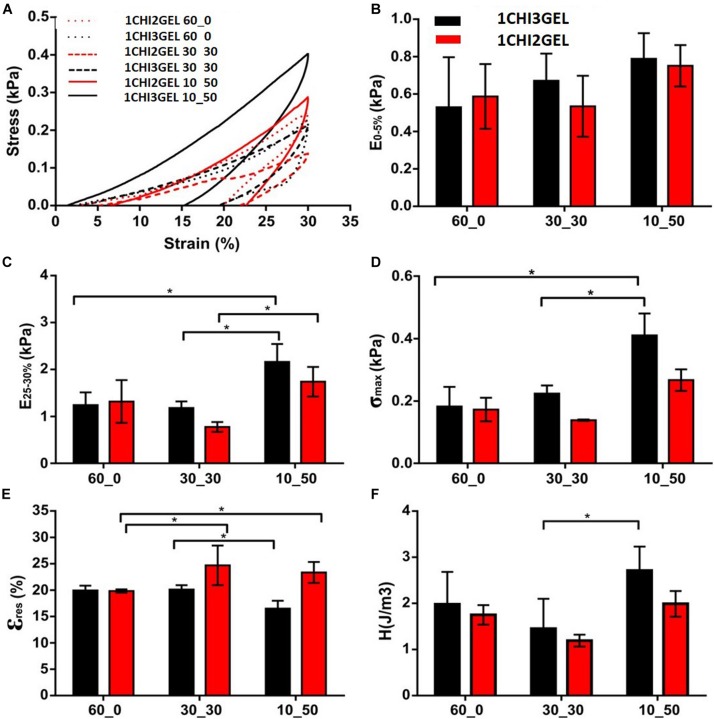
Mechanical characterization of 3D printed chitosan-gelatin scaffolds. **(A)** Representative stress-strain curves obtained for the 60_0, 30_30, 10_50 crosslinking conditions, and for the considered ratio (1CHI2GEL and 1CHI3GEL). Mechanical parameters calculated from the curves: **(B)** elastic modulus, **(C)** stiffness, **(D)** maximum stress, **(E)** residual deformation and **(F)** hysteresis area (*p < 0.05).

Mechanical compression tests were performed on the printed samples for each ratio (i.e., 1CHI2GEL, 1CHI3GEL) and for each crosslinking condition (Table 1). Stress-strain curves (Figure 6A) were all characterized by a load phase, in which the stress increased until the maximum stress value (σ_max_), corresponding to the maximum applied strain (ε = 30%), and an unload phase, when the stress decreased gradually while removing the strain. The different behavior of the load and unload phase for the considered configurations, corresponding to the loss of energy during the mechanical test, is due to the viscoelastic nature of the blends constituting the scaffolds. The mechanical testing results were reported in Figure 6 for all the considered samples. The elastic modulus values (E_0__–__5__%_, Figure 6B) showed there was no significant difference (*p* > 0.05) between the crosslinking conditions or gelatin/chitosan ratio. Regarding the stiffness (E_25__–__30__%_, Figure 6C), the highest value was detected for the 1CHI3GEL and 10_50 crosslinking condition, with a significant difference (*p* < 0.05) compared to the 1CHI3GEL and both 60_0 and 30_30 conditions. The same trend was observed for the maximum stress (σ_max_, Figure 6D). Hence, higher concentration of gelatin in the 1_3 ratio (6 vs. 4% w/v in 1CHI2GEL ratio) and the longer crosslinking period at 37°C (*t* = 50 min vs. 0 and 30 min in 60_0 and 30_30, respectively) contribute to an increase in mechanical properties for 10_50 condition, compared to 60_0 and 30_30 crosslinking conditions. Considering the residual deformation values (Figure 6E), the lowest value, related to a higher elastic behavior of the scaffolds, was attained for the 1CHI3GEL crosslinked under the 10_50 condition. Hence, a higher ability to recover the deformation after the exerted compression was detected for 10_50 condition, with significant difference (*p* < 0.05) compared to the 1CHI3GEL 30_30 crosslinking condition. The hysteresis area (Figure 6F) of the 1CHI3GEL 10_50 was the highest (i.e., higher viscous behavior of the structure), with significant difference (*p* < 0.05) compared to the 1CHI3GEL 30_30 condition. This means that the energy loss during the compression test for the 1CHI3GEL of the 10_50 condition was higher. The compressive elastic modulus obtained in this study for the 3D printed samples is found to be in a range between 0.53 ± 0.27 kPa (for the 60_0 of 1CHI3GEL) and 0.79 ± 0.14 kPa (for the 10_50 of 1CHI3GEL). These results were expected, as hydrogels suffer from weak mechanical properties (Vedadghavami et al., 2017). For the purpose of this study, the obtained range has to be compared to the elasticity range of native anisotropic tissues, as 4.5–8 kPa in skin (Karimi and Navidbakhsh, 2014), 12 kPa in skeletal (Gilbert et al., 2010), and 10–15 kPa in myocardial tissues (Pok et al., 2013). Indeed, it is well known that 3D scaffolds substitute extracellular matrix (ECM), whose mechanical properties regulate cell behavior, in terms of proliferation and differentiation. The obtainment of values closer to the ones of the native tissues is mandatory to obtain a scaffold able to provide the proper mechanical stimuli for the desired application. As it can be observed from the elastic moduli (Figure 6B), the ones obtained in this study are lower compared to the ones of the anisotropic tissues previously mentioned, but in the same order of magnitude. Moreover, it has been already reported that soft hydrogels can be used for the regeneration of non-loading areas, or for soft tissue regeneration (i.e., skin) (Kim et al., 2007). Testing higher concentrations of the polymers constituting the blend and crosslinker agents (Bettadapur et al., 2016; Gattazzo et al., 2018), adding growth factors (Rutledge et al., 2014; Castro et al., 2015) or using a bioreactor (Gauvin et al., 2011; Heher et al., 2015) could help reaching the desired values. In addition, the cells seeded on the 3D printed structures may produce ECM, allowing to increase the mechanical properties of the scaffold.

### Effect of Crosslinking Conditions on Scaffold Dimensions, Fiber Size and Distance Between the Fibers

**FIGURE 7:**
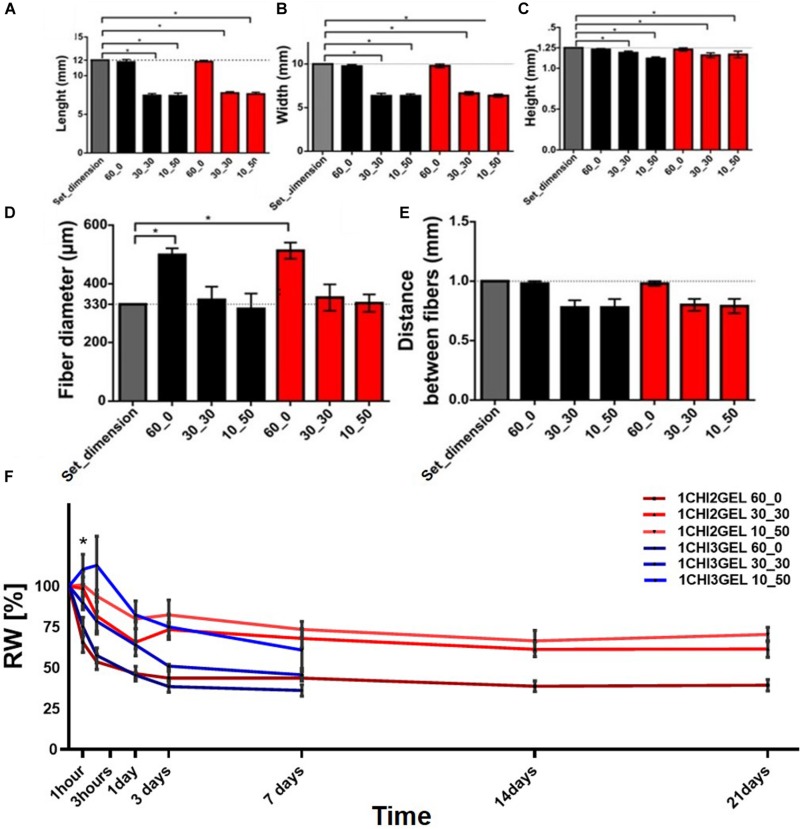
Printing accuracy and stability of 3D printed chitosan-gelatin scaffolds. The measurements have been acquired just after crosslinking time period. Dimensional values for each crosslinking condition with respect to **(A)** the length set dimension (12 mm), **(B)** width set dimension (10 mm), **(C)** height set dimension (1.25 mm). **(D)** Fiber diameter and **(E)** distance between fibers (compared to the set one, 1 mm) for each different crosslinking condition, **(F)** weight variation for both the ratio and the three crosslinking conditions considered in the study (*p < 0.05).

To determine the effect of different crosslinking conditions, overall dimensions (i.e., length, width, height), fiber diameter and distance between fibers of the printed samples were measured for each ratio (1CHI2GEL and 1CHI3GEL) and crosslinking condition (60_0, 30_30, 10_50). The differences in the overall scaffold dimensions can be qualitatively observed comparing the 60_0 condition to both 30_30 and 10_50 conditions (Figure 3). Chitosan/gelatin ratio (1CHI2GEL and 1CHI3GEL) did not cause any significant change (*p* > 0.05) in the scaffold dimensions, for any crosslinking conditions performed (Figures 7A–C). Scaffolds crosslinked under 60_0 condition had the closest dimensions to the CAD model (i.e., 98.4%, 97.9%, and 98.4% for length, width, height, respectively, Figures 7A–C), probably caused by the fact that these scaffolds were kept for all the crosslinking period at 4°C. In fact, at this temperature, gelatin is in a gel-like state, ensuring the maintenance of the 3D structure shape during the crosslinking as during the 3D printing process, in which the temperature of the base plate was set at 4°C. On the contrary, in the 30_30 and 10_50 crosslinking condition, the obtained values of length, width and height (Figures 7A–C) were statistically lower (*p* < 0.05) compared to the set ones and to the ones detected for 60_0 condition (i.e., 64.5%, 66.5%, 92.8% in 30_30 condition, and 62.1%, 63.8%, 93.6% in 10_50 condition, reported for length, width, height, respectively). This is due to the fact that, when samples are kept for 30 and 50 min at 37°C (in the 30_30 and 10_50 condition, respectively), gelatin is in a liquid-like state, resulting in the loss of shape and structural integrity (Piard et al., 2019). Indeed, as reported in a previous study (Dragusin et al., 2012), incubation at 37°C causes disintegration of the physical network of gelatin, due to the protein change from helix to coil above T_sol–gel_. Regarding fiber diameter and distance between fibers (Figure 7_D for fiber diameters, Figure 7_E for distance between fibers), for the fiber diameters obtained with the 60_0 condition (513.2 ± 27.4 μm), the dimensions are significantly different (*p* < 0.05) compared to the needle size dimension (ID = 330 μm), indicating a collapse of the fibers after the deposition. On the contrary, the fiber diameters obtained with the 30_30 (353.0 ± 44.9 μm) and 10_50 (334.1 ± 29.5 μm) conditions were closer to the needle size (*p* > 0.05), as consequence of the global reduction of the samples size in these conditions. Regarding the distance between the fibers, no significant difference was found (*p* > 0.05) for the considered crosslinking conditions, even if in 30_30 and 10_50 conditions the distance between the fibers was slightly decreased. Indeed, it is well known that cells have a different behavior on biomaterials composed of nano-scale architecture compared to micro-scale features (Kumbar et al., 2008). For instance, within anisotropic tissues, it has been reported that human skin fibroblasts show higher proliferation on fibers diameters in the range 350–1100 nm (Kumbar et al., 2008); for skeletal muscle tissue fiber diameter dimensions should be comprised in a range between 10 and 100 μm, to mimic as much as possible the diameter of adult muscle fiber, and 50–100 μm for the distance between the fibers (Cooper et al., 2010). Regarding myocardial regeneration, it has been shown that mimicking the hierarchical structure through the simultaneous deposition of microfibers (2–4 μm) and nanofibers (50–300 nm) favor cell-matrix interactions (Sreerekha et al., 2013). Comparing the values of the printed fiber diameters and distance between them obtained in this work, these are one order of magnitude higher than the ones desirable to mimic the native structure of anisotropic tissues and the ones obtained with other techniques [e.g., electrospinning (Kumbar et al., 2008; Cooper et al., 2010; Sreerekha et al., 2013), replica molding (Altomare et al., 2010) and soft lithography (Boldrin et al., 2007; Hwang et al., 2017)] used in anisotropic tissue regeneration. These techniques allow to obtain fiber diameter measurements at the nanometer scale, enhancing the substrate-cell interaction. Nonetheless, they did not fully satisfy mimicking native 3D tissue and manufacturing 3D constructs, being not able to reach and reproduce the real thickness value of anisotropic tissues [thickness of skin tissue ∼ 1–3 mm (Kumbar et al., 2008), skeletal muscle tissue ∼ 2–2.5 mm (Bian and Bursac, 2009), and myocardial tissue ∼ 1 cm (Sreerekha et al., 2013)]. On the contrary, 3D printing technology has the potential to build 3D constructs able to provide suitable microenvironment in which cells are spatially organized in 3D tissues, important requirement for the repair and regeneration of anisotropic tissues. Moreover, despite the 3D printing intrinsic limitations related to needle diameter size and selected printing parameters, very promising results for anisotropic tissues regeneration have been already reported (Lee et al., 2014; Costantini et al., 2017).

### Stability Test

Stability test was performed *in vitro* on the printed samples for each gel ratio (1CHI2GEL and 1CHI3GEL) and crosslinking condition (60_0, 30_30, and 10_50), maintaining the samples at 37°C up to 21 days (Figure 7F). Considering the same crosslinking condition, no significant difference (*p* > 0.05) was observed between the two gel ratios for the first 24 h. After 24 h, residual weight differences between the crosslinking conditions of both chitosan/gelatin ratio became significant (*p* < 0.05). In particular, the scaffolds of 1CHI2GEL samples show a swelling behavior (i.e., absorbing aqueous media) mainly between 24 h up to 3 days, followed by a progressive weight stabilization up to 21 days. The scaffolds of 1CHI3GEL after 24 h started to lose weight dramatically until complete disintegration of the 3D printed samples at 7 days.

When the stability test and mechanical testing results have taken together, we can assert the coexisting effects of bulk and surface erosion mechanisms (Storti and Lattuada, 2017). We hypothesize that in the early time points (during the first 48 h) the effect of surface erosion, which causes reduction in size without changing polymer structure properties, overcomes bulk erosion. Indeed, through the macroscopic observation of the 3D printed structures (Figures 3A–F), we can assess the change in dimensions. Moreover, when the stability has been further studied with SEM over 21 days, effect of surface erosion can clearly be seen at early time points while, at later time points, crumbling around the fibers starts, causing the 3D printed scaffolds losing their structural integrity (Supplementary Figures S4, S5). Moreover, the scaffolds have been evaluated over 21 days by FTIR analysis (Supplementary Figures S2, S3), and shift in the amide peaks indicated that the bulk erosion took place at later time points, when bulk erosion becomes dominant. Hence, mechanical properties evaluated at 48 h were not affected by bulk erosion effect, thus resulting higher in 1CHI3GEL. Over time, at later time points, the effect of chains cleaving due to bulk erosion on 1CHI3GEL becomes more dominant than in 1CHI2GEL, and this could be imputed to the higher presence of gelatin in 1CHI3GEL. Higher presence of gelatin is related with higher hydrophilicity and, thus, the effect of water penetrating the bulk cleaving hydrolytically chemical bonds bringing to the rupture of long chains into water-soluble fragments is higher (Rey-Vinolas et al., 2019). It has been reported that gelatin dissolution in presence of water is quicker when gelatin is in higher concentration (Kathuria et al., 2009; Nieto-Suárez et al., 2016), as in 1CHI3GEL. Although it has been observed that the presence of chitosan in a gelatin scaffold reduces its degradation rate, stabilizing the network, the influence of chitosan in our study was not sufficient to prevent gelatin loss in the 1CHI3GEL.

Comparing 60_0, 30_30, and 10_50 conditions, it can be observed that weight variation was not significant (*p* > 0.05) between 30_30 and 10_50 for any time point of the stability test. In 10_50, even a small swelling behavior was observed within the first hour (Huang et al., 2005). In particular, 1CHI2GEL 30_30 and 10_50 conditions show higher weight stability and no significant weight trend changes than 1CHI3GEL ones, mostly after 24 h. On the contrary, the difference between the 60_0 crosslinking condition and the other two (30_30 and 10_50) can be detected for all the considered timepoints for the both chitosan/gelatin ratios, specifically significant (*p* < 0.05) mainly within 3 h as the scaffolds tend to stabilize. The steep weight loss of the 60_0 condition compared to the others can be explained considering the crosslinking temperature of the samples. The samples in 60_0 were crosslinked at 4°C for 60 min at which gelatin was in its gel state (i.e., T = 25.5 ± 1°C for 1CHI2GEL, T = 26.4 ± 0.95°C for 1CHI3GEL). Thus, during crosslinking, gelatin was stable, the volume of the printed constructs was constant, and gelatin did not leach out from the structure. On the contrary, samples in 30_30 and 10_50 condition were at 37°C (above sol-gel temperature) during part of the crosslinking period, thus causing gelatin dissolution until stabilization. Proof of these considerations is the fact that the initial weight of the samples crosslinked with 30_30 and 10_50 conditions was lower than those of 60_0. Gelatin weight loss in the 3D samples was due to its temperature dependence, occurred as it was not crosslinked, in fact TPP only ionically crosslinked chitosan material.

In a previously reported study, using TPP as crosslinker for chitosan and gelatin blends, Yan et al. (2005) 3D printed tissue culture scaffolds with 0–90° orientation. In this study, the biomaterial ink composition has been chosen as 1:10 chitosan:gelatin and the crosslinking conditions was indicated as 3% (w/v) TPP for 5 min followed by glutaraldehyde crosslinking (0.25%) to further stabilize the structure. Authors have shown the stability of the constructs over 14 days, with cytocompatible characteristics (Yan et al., 2005). In comparison to the previously reported literature, the lower gelatin concentration in the blend, the higher TPP crosslinking concentration and TPP crosslinking time used in this study enabled us to create stable scaffolds up to 21 days in 0–180° orientation, without using additional crosslinkers.

### *In vitro* Cytocompatibility Test

**FIGURE 8:**
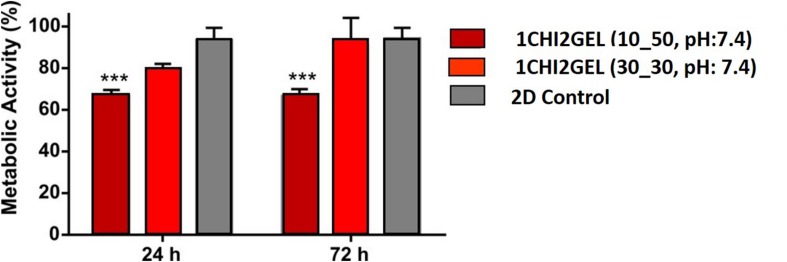
*In vitro* cytotoxicity test performed on the 1CHI2GEL scaffolds crosslinked under different crosslinking conditions (***p < 0.001).

For the *in vitro* cytocompatibility test, 1CHI2GEL crosslinked using 30_30 and 10_50 crosslinking conditions was tested. In fact, considering the results obtained in the stability test, only 10_50 and 30_30 crosslinking conditions of the 1CHI2GEL demonstrate good stability. Cell viability (Figure 8) was measured 66% at 24 h and 67% at 72 h for the 10_50 condition, and 79.7% at 24 h, to 91% at 72 h for the 30_30 condition, respectively, compared to the negative control group (i.e., 97%). This difference between 10_50 and 30_30 condition may be imputed to the higher gelatin diffusion to the media in the 30_30 condition, thus resulting in changes in the media composition. In the stability test, even if the residual weight percentages between these two groups is not statistically significant, lower residual weight of the scaffolds crosslinked under 30_30 condition can be noticed. In the first 24 h, the lower metabolic activity of the cells compared to the negative control group can be explained due to the leaching of gelatin to the media and changings in the composition of the media. However, over 72 h period it was observed that cells in contact with the scaffolds crosslinked 30_30 condition have shown comparable metabolic activity to negative control group while the cells in contact with the scaffolds crosslinked 10_50 condition have not shown any improvement regarding their metabolic activity. Hence, the best results were obtained with the 30_30 condition of the 1CHI2GEL, as they showed an increased cell viability at 72 h with no significant difference compared to the negative control. As a good interaction between the cells and the material is required by bioprinting, the mentioned condition was considered the optimal candidate for future 3D bioprinting experiment.

## Conclusion

In this study, chitosan-gelatin blend hydrogel was investigated as suitable bioink in 3D printing technology applications. The printing parameters (i.e., printing temperature, extrusion pressure, dispensing speed) were successfully optimized to obtain reproducible 3D printed anisotropic structures replicating the CAD design. Among the tested crosslinking conditions, chitosan/gelatin ratio, physico-mechanical and biological properties, 1CHI2GEL 30_30 was selected as the eligible formulation to be considered, thus paving the way for potential applications in anisotropic TE field. Briefly, for both CHI/GEL ratios, fiber diameters and distance between the fibers obtained in the 30_30 and 10_50 were found to be more suitable for future applications in anisotropic TE, although 60_0 crosslinking condition resulted the best in terms of shape retention. For all the crosslinking conditions and ratio tested, the obtained compression test values were of the same order of magnitude of the *in vivo* anisotropic tissue values. 3D printed scaffolds with 1CHI2GEL ratio and crosslinked with 30_30 and 10_50 conditions have shown better stability compared to all the conditions of 1CHI3GEL samples, as the latter disintegrated after 7 days. Therefore, 1CHI3GEL ratio was excluded from the study and it was not further considered in the cytocompatibility testing. Lastly, cell viability evaluated *in vitro* on 1CHI2GEL samples was higher in the 30_30 condition, compared to the 10_50 condition. In comparison to the previously reported studies, the lower gelatin concentration in the blend, the higher TPP crosslinking concentration and TPP crosslinking time used in this study enabled us to create stable cytocompatible scaffolds for 3D anisotropic tissue constructs.

## Data Availability Statement

All datasets generated for this study are included in the article/Supplementary Material.

## Author Contributions

NCe, NCo, and WS designed the study. TF and NCe performed the experimental work and analyses. TF, NCe, NCo, SF, and WS contributed to data discussion and wrote/edited the manuscript.

## Conflict of Interest

The authors declare that the research was conducted in the absence of any commercial or financial relationships that could be construed as a potential conflict of interest.
