# Supplementary material for: Tripolyphosphate-Crosslinked Chitosan/Gelatin Biocomposite Ink for 3D Printing of Uniaxial Scaffolds
Source: Front Bioeng Biotechnol. 2020 Apr 30;8:400. doi: 10.3389/fbioe.2020.00400 (PMC7203422; doi:10.3389/fbioe.2020.00400)
Supplement: Supplementary file 1 [file Data_Sheet_1.pdf]

Supplementary Material

Tripolyphosphate-crosslinked chitosan/gelatin biocomposite ink for 3D printing of uniaxial scaffolds

Tiziana Fischetti[1,2], Nehar Celikkin[1], Nicola Contessi Negrini[2,3], Silvia Farè[2,3], Wojciech Swieszkowski[1*]

[1] Faculty of Material Science and Engineering, Warsaw University of Technology, Warsaw, Poland
[2] Department of Chemistry, Materials and Chemical Engineering "G. Natta", Politecnico di Milano, Milan, Italy
[3] INSTM, National Consortium of Materials Science and Technology, Local Unit Politecnico di Milano, Milan, Italy

*** Correspondence:**
Prof. Wojciech Święszkowski
wojciech.swieszkowski@pw.edu.pl

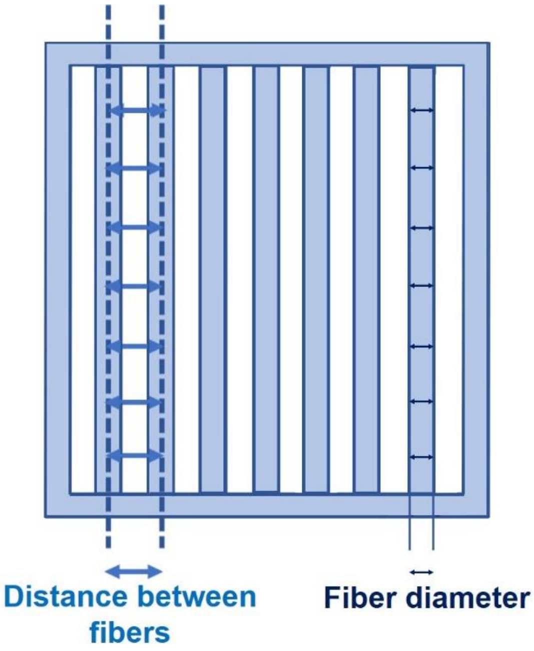

Figure S1. Measurement of fiber diameter and distance between fibers, calculated as the distance comprised between the axis of two adjacent fibers.

FTIR AND SEM characterization for an in-depth analysis of 3D structures

FTIR and SEM analysis were performed on 3D printed structures for each gel ratio (1CHI2GEL, 1CHI3GEL) and crosslinking condition (60_0, 30_30, 10_50) to investigate chemical and morphological modifications occurring over time to samples soaked in distilled water at 37°C. After the incubation period, 3D printed samples were lyophilized due to the fragile characteristic of the

scaffolds at late time points. For a thorough understanding of time-dependent modifications and to provide an in-depth analysis of the microscopic mechanisms determining the macroscopic process of degradation of the samples, a constant sampling rate was considered (1, 3, 5, 7, 10, 12, 14, 16, 18, 21 days). SEM analysis has only been performed for 1CHI3GEL scaffolds over 1, 3, 5 days as the samples lost their structural integrity (**Stability test, Par 3.5**) and we could not obtain fiber structures for SEM analysis. However, broken fragments of the fibers were lyophilized used for FTIR analysis over 21 days period, as the output spectra are not shape-dependent.

In the reported FTIR spectra, it can be observed a less pronounced peak at 3071 cm^{-1} (corresponding to gelatin amide A) mainly in later time points (already starting from 5 days) in each crosslinking condition of the 1CHI3GEL samples (**Figure S3, a), b), c)**) compared to 1CHI2GEL samples (**Figure S2, a), b), c)**). This behavior can be attributed to gelatin dissolution and leaching out of the 3D structure, that particularly occurs in 1CHI3GEL samples. Moreover, at 1540 cm^{-1} (corresponding to formation of amino groups due to chitosan/gelatin interaction in the blend) small shifts towards higher wavenumbers can be noticed in all the 3D samples regardless the ratio, more detectable in the 60_0 condition, i.e. the one with higher weight loss percentage (**Figure 7f**). Here as well, shifts in 1540 cm^{-1} peak are attributed to gelatin dissolution, but the effect results in all the ratio and conditions due to the chitosan/gelatin interactions in the blend. Moreover, chitosan characteristic peaks can be considered to further demonstrate gelatin dissolution. It has previously been reported (Sahraee et al., 2017) that chitosan peak at 3300 cm^{-1} becomes more intense when in higher content. This increasing peak trend over time is detectable for all the 3D samples in each ratio and conditions. This confirms that due to gelatin dissolution in water at 37°C, being this effect more significant in higher gelatin ratio (1CHI3GEL), chitosan remains as the main component of the 3D structure. Similar behavior and considerations can be observed also for 1030-1070 cm^{-1} and 1423-1465 cm^{-1} wavenumber ranges, that correspond to chitosan (Carvalho, 2017; Sahraee et al., 2017).

We attained valuable information also by SEM analysis, most specifically regarding how the diffusion of gelatin affects the fiber morphology at early time points and what happens when the scaffolds structurally disintegrate (**Figure S4, S5**). 1CHI2GEL scaffolds were structurally stable over the 21 days for each crosslinking condition, as reported in the main text. The main difference among the 60_0 scaffolds and other crosslinking conditions was the porosity of the surface due to the diffusion of gelatin. As discussed in the manuscript, the 60_0 scaffolds crosslinked with TPP at 4°C were structurally intact with the set fiber and scaffold dimensions. However, when the stability test started, the gelatin started to diffuse out from the crosslinked chitosan fibers, which resulted in the porosity on the fibers. This effect has been observed significantly less in other crosslinking conditions because when the scaffolds were transferred to 37 °C for a period during the crosslinking, chitosan fibers managed adapt to take over the pores created due to the leaching of gelatin. At later time points, we have observed that crumbling on the fibers has occurred due to degradation (**Figure S4**). Regarding the 1CHI3GEL scaffolds (**Figure S5**), we have also observed surface porosity for 60_0 scaffolds; however, this was not as significant as 1CHI2GEL scaffolds. In the images it can be clearly seen that the crumbling for these fibers starts on day 3 and progressively increases on day 5, and after this time point it was not possible to attain structurally stable fibers for the SEM analysis.

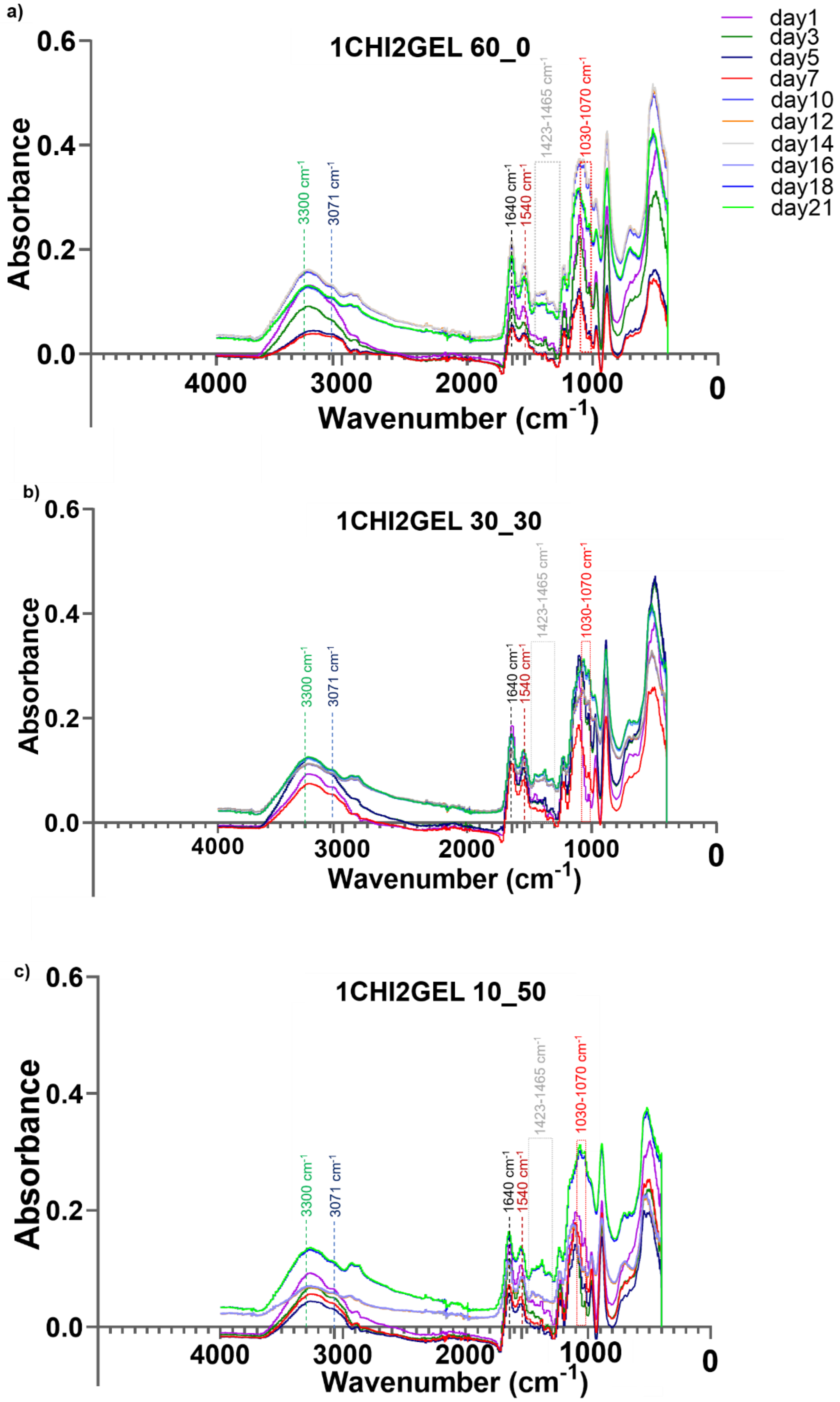

Figure S2. FTIR spectra for each crosslinking conditions (60_0, 30_30, 10_50) of 1CHI2GEL 3D structures at different time points (1, 3, 5, 7, 10, 12, 14, 16, 18, 21 days). (a) 1CHI2GEL 60_0 3D printed structure, (b) 1CHI2GEL 30_30 3D printed structure, (c) 1CHI2GEL 10_50 3D printed structure.

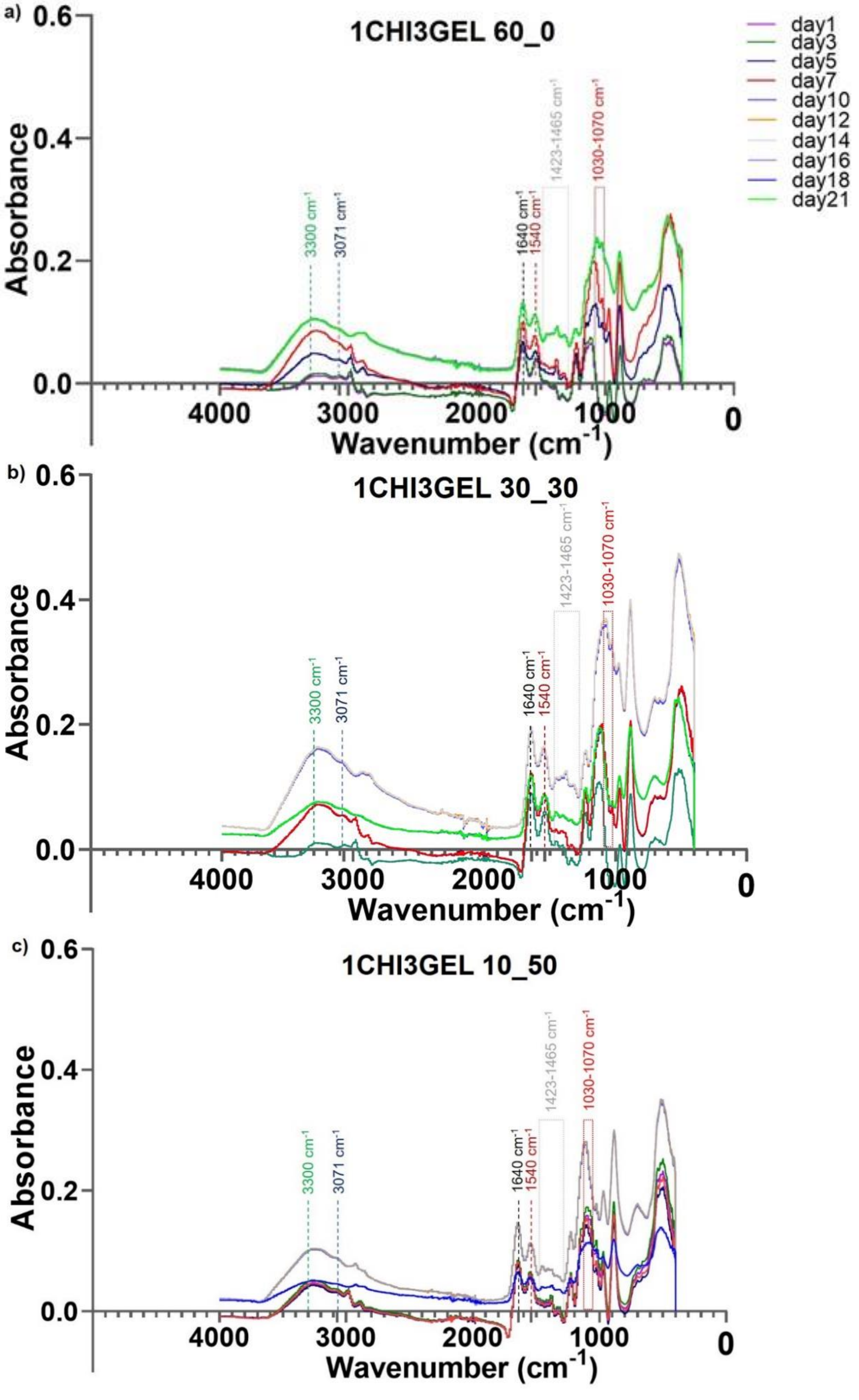

Figure S3. FTIR spectra for each crosslinking condition (60_0, 30_30, 10_50) of 1CHI3GEL 3D printed structures at different time points (1, 3, 5, 7, 10, 12, 14, 16, 18, 21 days). (a) 1CHI3GEL 60_0 3D printed structure, (b) 1CHI3GEL 30_30 3D printed structure, (c) 1CHI3GEL 10_50 3D printed structure.

1CHI2GEL

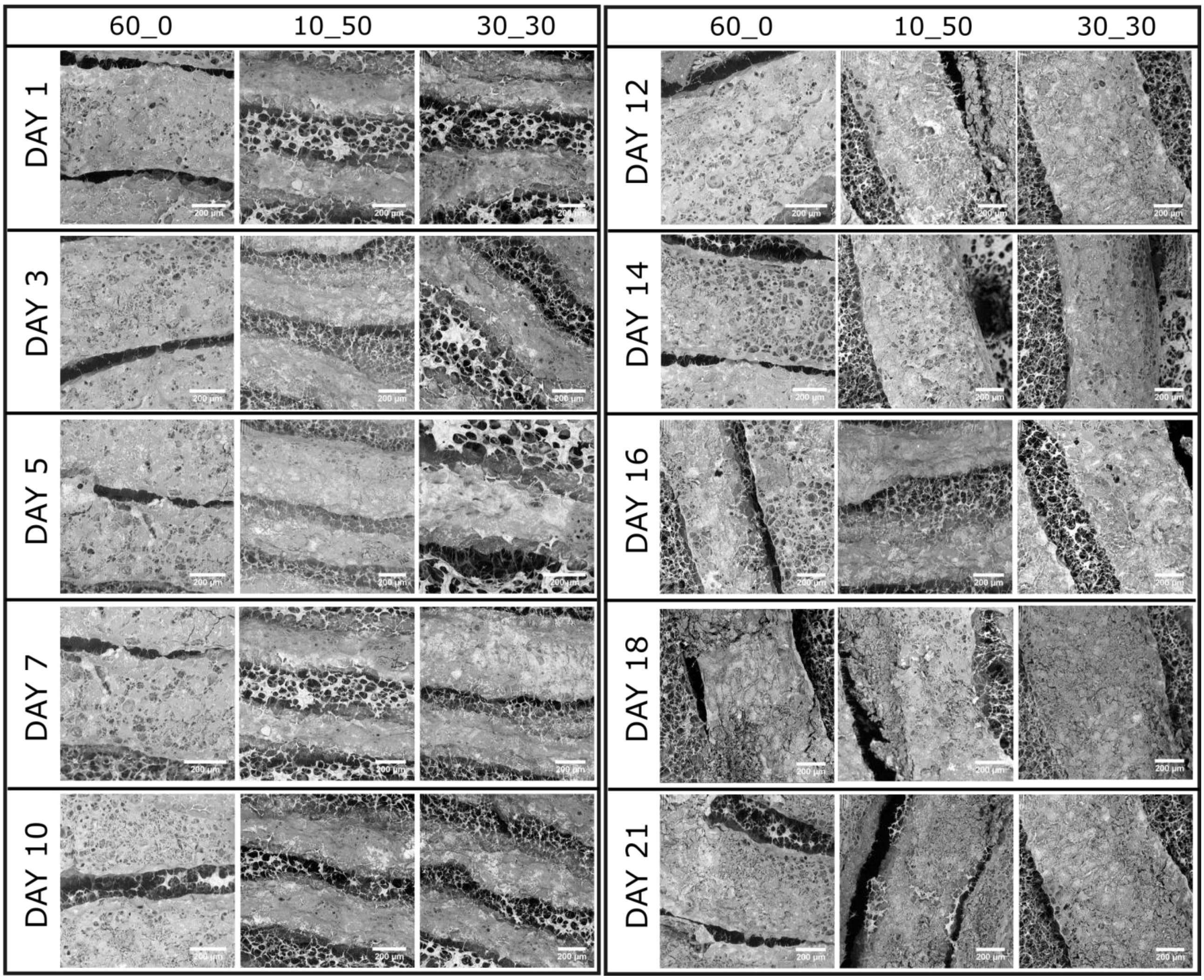

Figure S4. SEM images of fibers for each crosslinking conditions (60_0, 30_30, 10_50) of 1CHI2GEL 3D structures at different time points (1, 3, 5, 7, 10, 12, 14, 16, 18, 21 days). (Scale bar: 200µm).

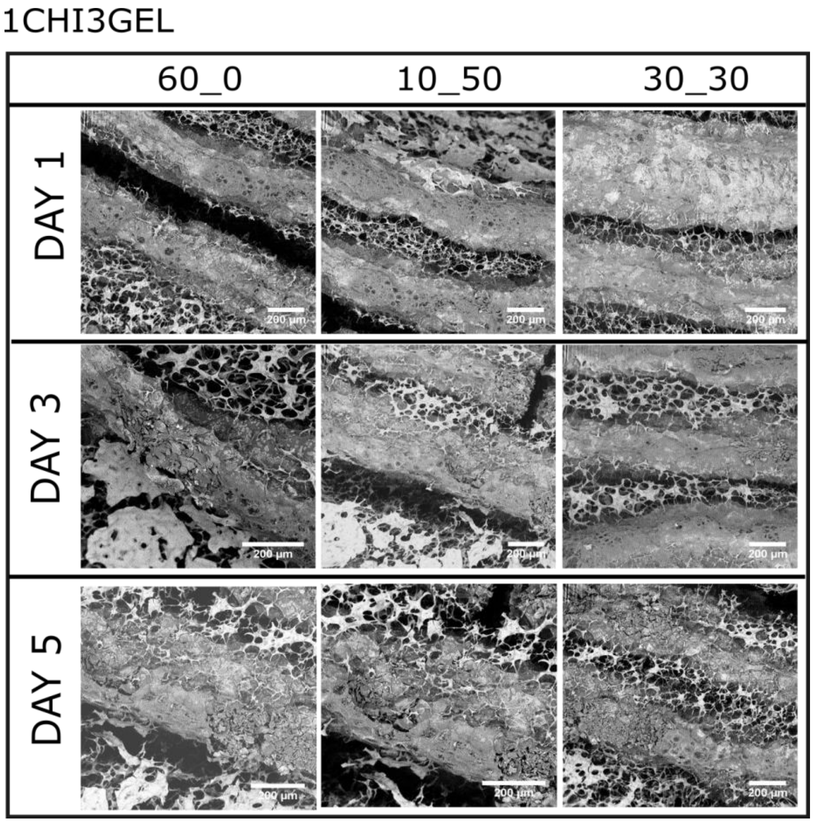

Figure S5. SEM images of fibers for each crosslinking conditions (60_0, 30_30, 10_50) of 1CHI3GEL 3D structures at different time points (1, 3, 5 days). (Scale bar: 200μm).

Reference

Carvalho, I. C. (2017). Engineered 3D-scaffolds of photocrosslinked chitosan-gelatin hydrogel hybrids for chronic wound dressings and regeneration. *Materials Science and Engineering C*, *78*, 690–705. https://doi.org/10.1016/j.msec.2017.04.126

Sahraee, S., Milani, J. M., Ghanbarzadeh, B., & Hamishehkar, H. (2017). Physicochemical and antifungal properties of bio-nanocomposite film based on gelatin-chitin nanoparticles. *International Journal of Biological Macromolecules*, *97*, 373–381. https://doi.org/10.1016/j.ijbiomac.2016.12.066
